# Reducing Radiation Exposure to Paediatric Patients Undergoing [18F]FDG-PET/CT Imaging

**DOI:** 10.1007/s11307-021-01601-4

**Published:** 2021-04-12

**Authors:** Hunor Kertész, Thomas Beyer, Kevin London, Hamda Saleh, David Chung, Ivo Rausch, Jacobo Cal-Gonzalez, Theo Kitsos, Peter L. Kench

**Affiliations:** 1 QIMP Team, Center for Medical Physics and Biomedical Engineering, Medical University of Vienna, Währinger Gürtel 18-20, 1090 Vienna, Austria; 2 Department of Nuclear Medicine, The Children’s Hospital at Westmead, Sydney, NSW Australia; 3 Faculty of Medicine and Health, Discipline of Child and Adolescent Health, Children’s Hospital Westmead Clinical School, The University of Sydney, Sydney, NSW Australia; 4 Ion Beam Applications, Protontherapy Center Quironsalud, Madrid, Spain; 5 Discipline of Medical Imaging Science and Brain and Mind Centre, Faculty Medicine and Health, The University of Sydney, Sydney, NSW Australia

**Keywords:** PET/CT, Paediatric imaging, Radiation exposure, Low count imaging, Image reconstruction

## Abstract

**Purpose:**

To investigate the possibility of reducing the injected activity for whole-body [18F]FDG-PET/CT studies of paediatric oncology patients and to assess the usefulness of time-of-flight (TOF) acquisition on PET image quality at reduced count levels.

**Procedures:**

Twenty-nine paediatric oncology patients (12F/17M, 3–18 years old (median age 13y), weight 45±20 kg, BMI 19±4 kg/m^2^), who underwent routine whole-body PET/CT examinations on a Siemens Biograph mCT TrueV system with TOF capability (555ps) were included in this study. The mean injected activity was 156 ± 45 MBq (3.8 ± 0.8 kg/MBq) and scaled to patient weight. The raw data was collected in listmode (LM) format and pre-processed to simulate reduced levels of [18F]FDG activity (75, 50, 35, 20 and 10% of the original counts) by randomly removing events from the original LM data. All data were reconstructed using the vendor-specific e7-tools with standard OSEM only, with OSEM plus resolution recovery (PSF). The reconstructions were repeated with added TOF (TOF) and PSF+TOF. The benefit of TOF together with the reduced count levels was evaluated by calculating the gains in signal-to-noise ratio (SNR) in the liver and contrast-to-noise ratio (CNR) in all PET-positive lesions before and after TOF employed at every simulated reduced count level. Finally, the PSF+TOF images at 50, 75 and 100% of counts were evaluated clinically on a 5-point scale by three nuclear medicine physicians.

**Results:**

The visual inspection of the reconstructed images did not reveal significant differences in image quality between 75 and 100% count levels for PSF+TOF. The improvements in SNR and CNR were the greatest for TOF reconstruction and PSF combined. Both SNR and CNR gains did increase linearly with the patients BMI for both OSEM only and PSF reconstruction. These benefits were observed until reducing the counts to 50 and 35% for SNR and CNR, respectively.

**Conclusions:**

The benefit of using TOF was noticeable when using 50% or greater of the counts when evaluating the CNR and SNR. For [18F]FDG-PET/CT, whole-body paediatric imaging the injected activity can be reduced to 75% of the original dose without compromising PET image quality.

**Supplementary Information:**

The online version contains supplementary material available at 10.1007/s11307-021-01601-4.

## Introduction

Since the introduction of the first combined PET/CT systems in the late 1990s [1], this hybrid imaging modality has become well established for paediatric imaging [2]. However, both PET and CT are associated with ionising radiation. In the case of PET, radiation exposure is directly proportional to the injected tracer activity. According to the Euratom Directive, Article 67, the ALARA (‘As Low as Reasonably Achievable’) principle should be followed for imaging examinations involving ionizing radiation and requires the adaptation of the injected dose to the lowest level compatible with adequate image quality that provides sufficient clinical information [3]. In that regard, younger patients and patients who undergo repeated PET scans should benefit from applying ALARA so as to limit the total radiation exposure [4]. To follow the ALARA principle, the European Association of Nuclear Medicine (EANM) issued the paediatric dosage card in 2008 [5–8]. The guidelines established by the EANM [6] and the North American consensus guidelines [4] were harmonized in 2014 [8]. Despite the unquestionable desirability of low radiation exposure, a reduction of the injected tracer amounts inevitably causes increased noise levels in the reconstructed PET images. Furthermore, PET image quality strongly depends on the acquisition time, reconstruction and correction schemes and system specifications.

To overcome these limitations, a wide range of technical PET developments—both in software and hardware—was translated from research and development into clinical systems. Compared with the prototype system, today’s clinical PET systems are equipped with lutetium orthosilicate (LSO) or LYSO crystals with improved coincidence timing resolution [9]. Currently, all clinical PET/CT systems are equipped with time-of-flight (TOF) capability [10] with a timing resolution varying between about 200-500 ps [9, 11, 12]. Several studies did evaluate the use of TOF in various oncology studies [13–19] and demonstrated a benefit of incorporating TOF information into the image reconstruction process for lesion detection, particularly in larger patients [11, 20]. Different groups studied also the feasibility of reducing the injected [18F] FDG activity levels; however, the number of studies on low-dose paediatric PET imaging is limited [21–24].

In this study, we investigate the effects of reducing the injected [18F]FDG activity in paediatric oncology patients undergoing whole-body PET/CT examinations. Specifically, the effect of TOF was evaluated for different image reconstruction methods at low count levels. We also involve clinical readers to assess the quality of the reconstructed PET images at different dose levels.

## Materials and Methods

### Patient Acquisitions

This study was performed retrospectively (Ethics number 2019/ETH00138). It included 29 paediatric patients (12 females, 17 males, 3–18 years old (median age 13y), weight 45 ± 20 kg, BMI 19 ± 4 kg/m^2^), who underwent routine whole-body [18F]FDG PET/CT examinations. All patients were administered [18F]FDG, and the injected activity was scaled according to the patients weight based on a maximum activity of 200 MBq for a patient weighing 70 kg or above. Image acquisition was performed approximately 45–60 min after tracer injection. The mean injected activity was 156 ± 45 MBq (activity concentration 3.8 ± 0.8 MBq/kg). The details of the individual patients including diagnosis are summarized in Table 1.

**Table 1:** Patient demographics including the diagnosis and the location of the lesions for subsequent SUV-based evaluation

Patient name	Age [years]	Gender	Weight [kg]	Height [m]	Body mass index [kg/m^2^]	Injected FDG activity [MBq]	Activity concentration [kg/MBq]	Diagnosis	Lesions (SUVmax)	# lesions
P1	8	M	29	1.3	16.2	136	4.68	Stage 1 Hodgkins lymphoma	Cervical level 2 lymph nodes bilaterally, left (2.1) and right (1.7)	2
P2	8	M	26.7	1.3	16.6	124	4.64	Cardiac myxofibrosarcoma	Tumour surrounding aortic valve	1
P3	15	F	48.9	1.6	19.6	188	3.85	Giant cell tumour of the right maxilla	Giant cell tumour right maxilla (2)	1
P4	17	F	70.2	1.7	24.0	210	2.99	Metastatic osteosarcoma	Left inguinal lymph nodes (2.4), left external iliac node (2.8)	2
P5	6	F	21.4	1.1	17.1	102	4.77	Post lung transplant lymphoma	Left hilum (3.2), left lung mid zone (3)	2
P6	17	M	77.4	1.9	21.4	205	2.65	Burkitts lymphoma	-	-
P7	15	M	57.6	1.7	21.2	185	3.20	Osteoblastic osteosarcoma	Right humerus (10.2), right iliac crest anteriorly (5.5), sixth left rib posteriorly (3)	3
P8	15	M	49.4	1.7	18.1	172	3.48	Burkitts Lymphoma	The cervical lymph nodes (2), left axilla node (2.1)	2
P9	15	F	46.4	1.5	19.6	179	3.85	Sarcoma of chest wall	the right lung pleural Thickening (2.2), the right lobe of liver (5.4)	2
P10	14	F	70.5	1.7	25.9	201	2.85	Ewing sarcoma	Costal elements of S2 and S3 (8.9), T9 vertebral lesion (13.9), L4 vertebra (4.2)	3
P11	18	M	44.7	1.7	14.9	159	3.57	Hepatcellular carcinoma	-	-
P12	10	M	29.7	1.4	15.8	121	4.06	Osteosarcoma	Cervical lymph nodes	1
P13	14	M	69	1.7	22.8	195	2.83	Burkitts lymphoma	Thymus	1
P14	15	F	60.1	1.6	24.7	208	3.46	Hodgkins lymphoma	Right axillary nodes (2.3 - 3.4) and in the right supraclavicular node (3.6)	2
P15	13	F	73.8	1.7	25.2	216	2.92	Osteosarcoma	Right inguinal lymph node (7.4), right external iliac lymph nodes anterior node (5.4) and posterior pelvic sidewall node (6.6)	3
P16	12	M	44.1	1.3	28.2	148	3.37	Prune Belly syndrome with renal impairement	Large bovel	1
P17	6	F	23.7	1.2	17.3	93	3.92	LCH	-	-
P18	15	M	50.1	1.8	15.6	177	3.54	Hodgkins disease IIa	-	-
P19	15	M	65.6	1.8	19.4	211	3.22	Lymphoma	-	-
P20	13	M	43.3	1.6	17.3	160	3.70	Arthritis	-	-
P21	16	F	69.6	1.7	25.6	197	2.84	Hodgkins lymphoma	-	-
P22	12	M	46.8	1.7	17.2	184	3.94	Osteosarcoma (right distal femur)	The left lung- apical left lung (2.7), left lower pleural based anterior lesion(2.7) and at the left hilum (2) and right lung anterior cardiophrenic lesion (2.6), right femur and prosthesis(4.2),	2
P23	7	F	21	1.3	12.6	106	5.04	B-cell lymphoblastic lymphom	-	-
P24	7	M	26.6	1.3	14.8	110	4.12	RMS left flexor hallicus longus muscle	Left calf flexor hallucis longus (2.3)	1
P25	12	F	32	1.4	16.8	118	3.69		Parietal lesion, the left lateral clavicle (2)	1
P26	4	M	21	1.1	19.0	106	5.06	LCH right mastoid	-	-
P27	2	F	10	0.8	16.0	58	5.81	LCH with multi-system, multifocal disease	Left proximal femur (2.6), left proximal humerus (1.43)	2
P28	3	M	16.9	1.0	15.9	87	5.17	LCH	-	-
P29	17	M	51.9	1.8	16.8	173	3.34	Osteosarcoma	C2 left cervical mass (6.7), T4 vertebral body (4.8) and right proximal humerus (5)	3

### Optimized Image Reconstruction Protocol

All patients were scanned on a Siemens Biograph mCT TrueV (Siemens Medical Solutions, Knoxville, TN, USA) PET/CT system with an axial field-of-view of 21.8 cm with 555 ps TOF resolution [25]. Emission scans were performed for 2 min per bed position. The raw data was collected in list-mode (LM) format and reconstructed into PET images with a matrix size of 200 x 200 and a voxel size of 4.073 x 4.073 x 2.027 mm^3^. A low-dose CT (100 kVp, reference tube current time product: 80 mAs, slice thickness: 3mm) scan was performed for the purpose of CT-based attenuation correction. The matrix size for the CT images was set to 512 x 512 with a voxel size of 0.976 x 0.976 x 3.0 mm^3^.

PET image reconstruction was performed using Siemens e7tools (Siemens Medical Solutions, Knoxville, TN, USA). Three patient studies with multiple lesions were randomly selected to help define the optimum image reconstruction parameters with TOF (TOF) and without incorporating the TOF (non-TOF) information. These studies were reconstructed with iterations from 1 to 8, with 14 subsets and with a 5mm full-width-half-maximum (FWHM) Gaussian post-filter. For the evaluations, spherical volumes-of-interest (VOIs) were placed in the background region (measured in the liver) and in the lesions.

The lesion VOIs were defined individually for every patient by manually placing the VOI in the tumour. The diameter of the VOI was then adjusted to best match the metabolic lesion volume as appearing in the reconstructed PSF+TOF PET images. The size of the VOI in the background region was fixed with a diameter of 20 mm.

The convergence of the different image reconstruction algorithms was defined by measuring the contrast [13]:

1 $$ Contrast=\frac{Mean_{lesion}}{Mean_{background}} $$

where the *Mean*_*background*_ was calculated as the mean value within the VOI placed in the background region (liver) and the *Mean*_*lesion*_ is the mean values within the VOI placed in the evaluated lesion.

The mean standard uptake value (SUV_mean_) was calculated as:

2 $$ {SUV}_{mean}=\frac{Act\mathrm{i}{vity}_{VOI}}{\frac{{ Act ivity}_{injected}}{Bodyweight}} $$

where the Activity_VOI_ is the mean value calculated within the VOI placed in the lesions (given in kBq/ml), Activity_injected_ is the administered activity to the patient (in MBq) and the Bodyweight is the weight of the patient (kg). In addition, noise properties of the images were evaluated for defining the suitable reconstruction parameters:

3 $$ Noise=\frac{SD_{background}}{Mean_{background}} $$

The image contrast as a function of the number of iterations and the SUV_mean_ as a function of the image noise were analysed. These curves were calculated for different lesions, including lung lesions located in the left hilum, lung tumour and a lesion located in the iliac crest. Furthermore, the reconstructed images were visually assessed by two nuclear medicine physicians to define the optimal reconstruction parameters.

Following the initial evaluation, the data were reconstructed with four different methods the standard 3D Ordinary Poisson ordered subsets expectation maximisation algorithm (OSEM only), OSEM with time-of-flight (TOF), OSEM with point spread function (PSF) [26] and finally PSF with TOF (PSF+TOF). To all reconstructed images, a 5-mm FWHM Gaussian post-filter was applied. The combination of count levels and reconstruction techniques evaluated is summarised in Supplementary Figure 1.

### Virtual Dose Reduction

To simulate the reduction of the injected dose, the original LM data were pre-processed by randomly deleting events in the original LM data before the actual image reconstruction. The adapted version of the RANECU subroutine [27] was modified to give a single random number at each call. The selection criterion of an event was based on the percentage of the original counts to be stored in the new LM file and normalised to a value between 0 and 1. The schematic workflow for achieving 50% of the actual counts is presented in Supplementary Figure 2. If the corresponding random number for an event *i* was found to be larger than the threshold used for selection criteria (*S*), then the event was deleted from the LM data. Otherwise, the event was stored in a new list-mode file with reduced counts, together with the time and tag events.

This reduction of counts is equivalent to a ‘virtual’ reduction of the injected dose by a factor (*1/S*). Assuming that the activity level is far from the noise equivalent count rate (NEC) peak, then prompts can be approximated by a linear function since both the quadratic behaviour of the random counts and the dead time effects are negligible. A simulated injected activity of 75, 50, 35, 20 and 10% was applied in the current study.

### Quantitative Image Analysis

The image analysis was done for every count rate and image reconstruction combination using three quantitative figures-of-merit. First, the Contrast (Eq. 1) and Noise (Eq. 3) were calculated. The images were evaluated for signal-to-noise ratio (SNR) defined as:

4 $$ SNR=\frac{Mean_{VOI}}{SD_{VOI}} $$

where Mean_VOI_ is the mean value in the VOI and SD_VOI_ is the standard deviation in the same VOI. The effect of TOF on the reconstructed images was evaluated by calculating the TOF gain as:

5 $$ {SNR}_{gain}=\frac{SNR_{TOF}}{SNR_{non- TOF}} $$

The contrast-to-noise ratio (CNR) was calculated as the difference between the mean value in the lesion and the mean value in the background region calculated in the liver, divided by the standard deviation in the background region [28]:

6 $$ CNR=\frac{Mean_{lesion}-{\mathrm{M} ean}_{background}}{SD_{background}} $$

The CNR gain was calculated for all 4 reconstructions: OSEM only, TOF, PSF and PSF+TOF. All patients were analysed for image noise and SNR gain (measured in the liver) and CNR gain for all the identified lesions. Further information is given in Table 1.

### Clinical Image Quality Assessment

The review of the reconstructed clinical images was based on the reconstruction protocols used in the clinic routinely: PSF+TOF with a 5-mm FWHM Gaussian post-filter. Images were reconstructed using raw emission data at 50, 75 and 100% of the original counts. The images were anonymised, mixed and randomly grouped into three groups for the evaluation. Every reading day consists of 29 patients (a patient was only shown once per reading day) in a random order (subjects and count levels). The schematic of the clinical evaluation is summarised in Supplementary Figure 3. In total, 87 data sets were presented to the reader using Radiant Medical Imaging (Radiant Medical Imaging, Scarborough, ON, Canada) image visualisation software [29].

Three experienced nuclear medicine physicians, each with more than 10-year experience as a paediatric nuclear medicine specialist, were engaged in the review of the clinical images. The patient age and indication for the scan was available to the reading physician and each reading session was separated by a minimum one-week time period to minimise the possibility to remember the presented images from the previous week.

For every patient, four quality questions had to be answered:

- What is the overall image quality?
- How would you rate image noise?
- How would you rate image smoothness?
- How would you rate your ability to detect lesions based on the scan?

by giving a score on a 5-point scale: 1, very poor/unacceptable; 2. poor/unacceptable; 3, suboptimal/acceptable; 4, adequate/acceptable; and 5, optimal.

The results were compared patient by patient at different count levels, and the significance of the evaluation at different count levels was evaluated statistically by first analysing the distribution of the collected data using the D’Agostino-Pearson normality test. Then, a Pearson’s correlation analysis was done reporting the coefficient of correlation (r) and the respective *p* values. All statistical evaluations were done using GraphPad Prism 8.0 Software (GraphPad Software, San Diego, California USA) [30]. The results were evaluated individually for each reader comparing 100% with 75 and 50% for all four questions (image quality, noise, image smoothness and lesion detectability) as well as the average of the three readings.

### Injected Activity Levels

The virtual activity levels were calculated for every patient following the EANM paediatric dosage card and compared with original injected activities. The EANM recommendation is defined as a baseline activity for every isotope and examination type multiplied with the weight-based multiple, which is also dependent on the class of the examination. In the case of 18F[FDG] torso examinations, the recommended baseline activity is 25.9 MBq, and it is categorised as class B with a maximum recommended injected activity of 362.6 MBq for a patient with 68 kg weight. Following the North American consensus recommendations, the minimum injected activity is also set to 26 MBq, and the injected activity is calculated as weight-based to range between 3.7 and 5.2 MBq/kg. This would correspond to an injected activity of 353.6 MBq for the same 68 kg patient as described above (calculated with 5.2 MBq/kg).

## Results

### Optimized Image Reconstruction Protocol

**Fig. 1.**
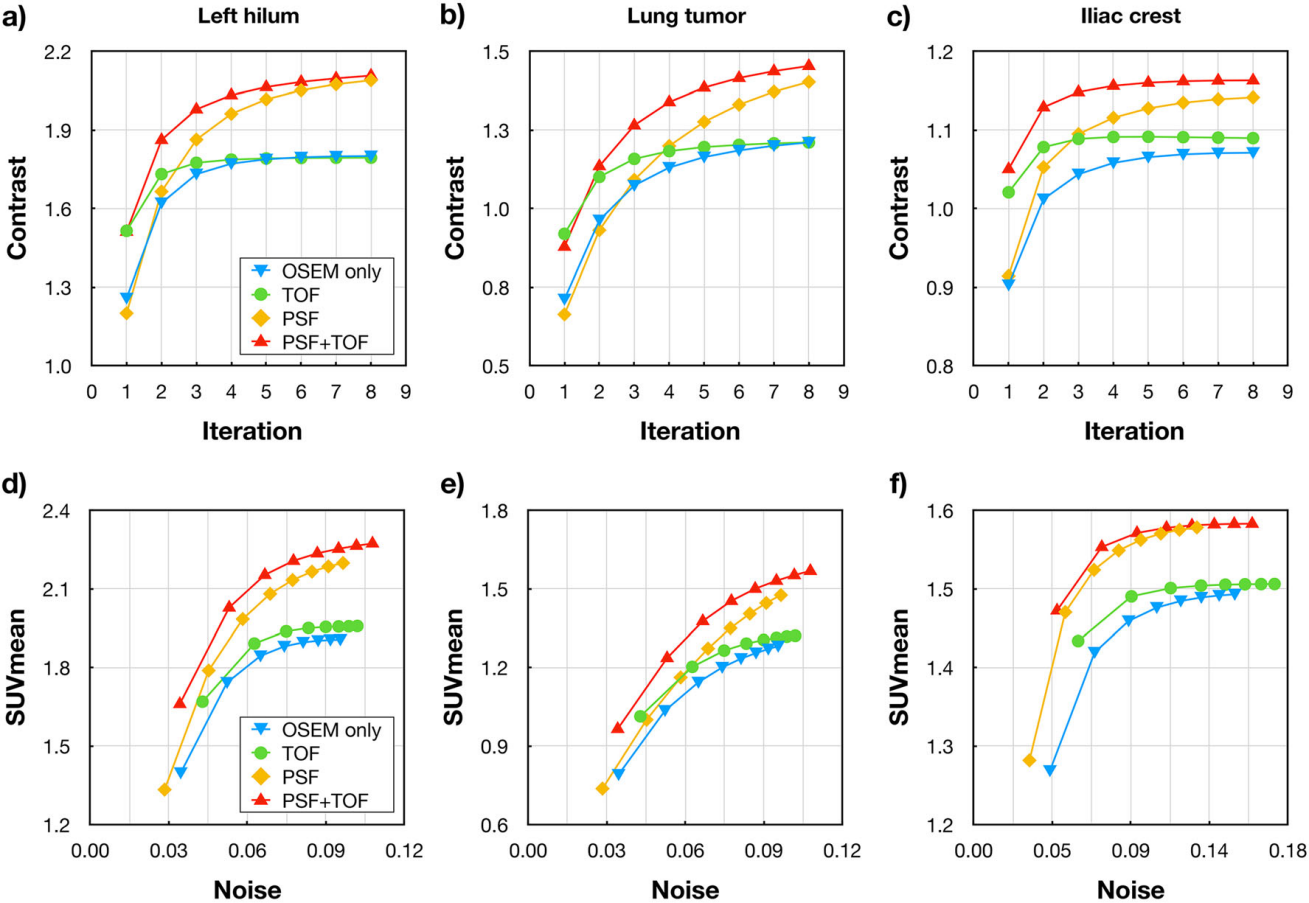
Convergence analyses of the applied image reconstruction techniques. Every data point corresponds to one iteration (1–8) with 14 subsets. Different lesions of selected patients were evaluated: (**a**–**d**) lesion in the left hilum (Patient 21), (**b**–**e**) lung tumour (Patient 21), (**c**–**f**) tumour located in the humerus (Patient 23). For the non-TOF reconstruction (blue, OSEM only, and yellow, PSF) 5 iterations with 14 subsets were selected, while 3 iterations with 14 subsets were chosen for TOF reconstructions (green , TOF, and red, PSF+TOF).

First, suitable image reconstruction settings were determined. Fig. 1 shows the convergence curves of the contrast as a function of the iteration number and image noise. In lung lesions, the similar image contrast was achieved with 5 iterations (14 subsets) for OSEM only and with 3 iterations (14 subsets) for TOF reconstructions. Differences became more pronounced for lesions located in the iliac crest, where 6 iterations were required to obtain the same level of contrast. With the increased number of iterations to match contrast for the TOF and OSEM only (non-TOF) reconstruction, the noise levels were significantly increased. Based on the analysed curves for the two lung lesions and the tumour located in the humerus, 5 iterations and 3 iterations were chosen for the non-TOF (OSEM only and PSF) and for TOF reconstruction (TOF and PSF+TOF), respectively. All reconstructions used 14 subsets and a 5-mm FWHM Gaussian post-filter.

### Quantitative Image Analysis

**Fig. 2.**
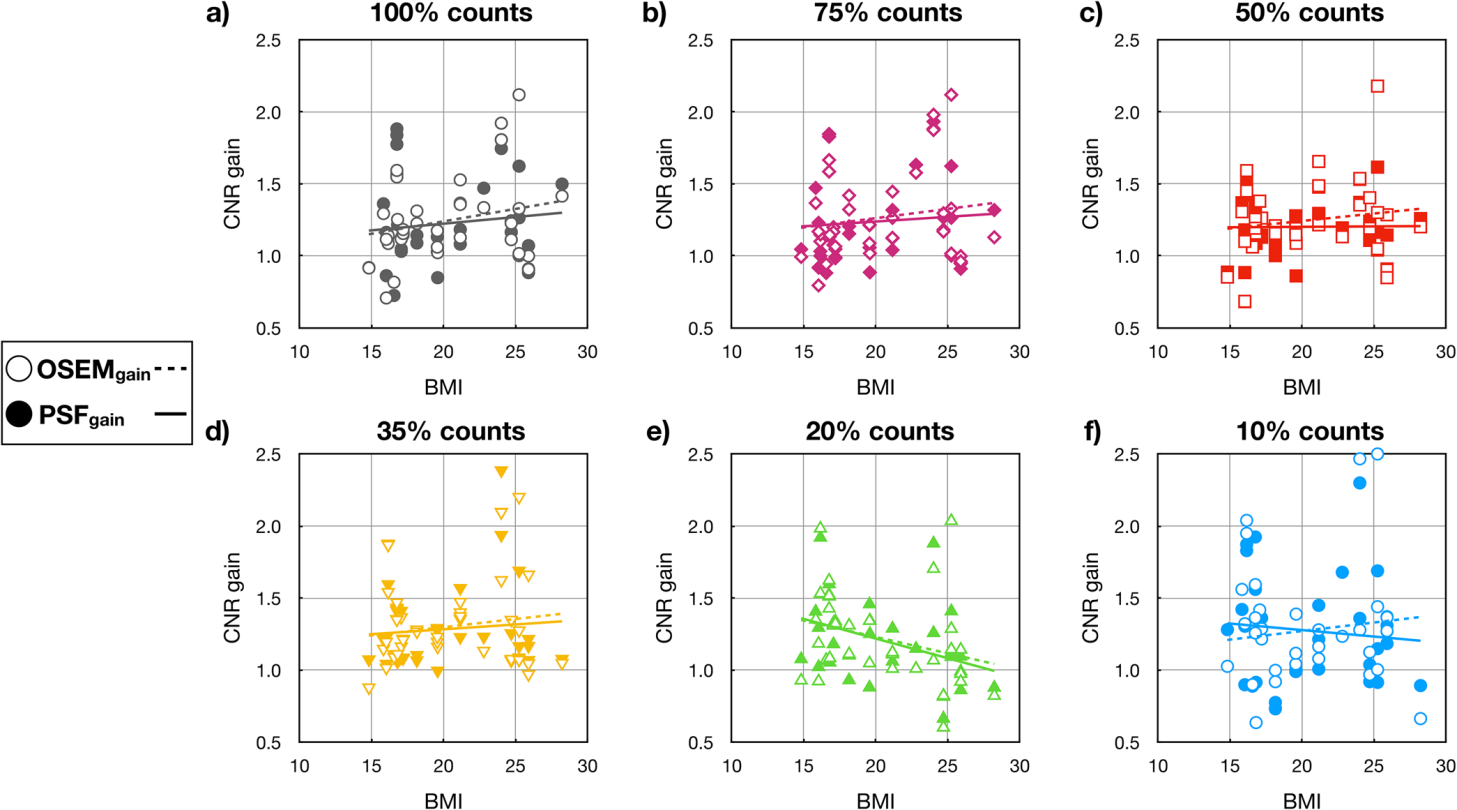
CNR gain of the 35 analysed tumours as a function of the BMI at all the count levels: 100, 75, 50, 35, 20 and 10% (**a**–**f**) of the original counts. Empty symbols correspond to OSEM CNR gain (OSEM only vs. TOF) and solid symbols to the PSF CNR gain (PSF vs. PSF+TOF). The gain in the CNR is lower when PSF is included in the reconstructions. The trend is shown as a linear regression with solid lines for PSF CNR gain reconstructions and dashed line for the OSEM CNR gain.

In total, 35 lesions were analysed (6 head and neck, 8 in the thorax region, 6 lung tumours, 9 in the abdomen and 6 in the extremities). The highest CNR gains were 2.1, which were calculated for tumours in the abdomen of patients with a BMI of 24 reconstructed with OSEM only. At 100% of counts, no CNR gain was noticed for 5/35 delineated lesions. The same was true for lower simulated count levels (Fig. 2). CNR gain increased slightly with BMI until 35% of the counts. Below this level of counts, the distribution of gains became unpredictable.

**Fig. 3.**
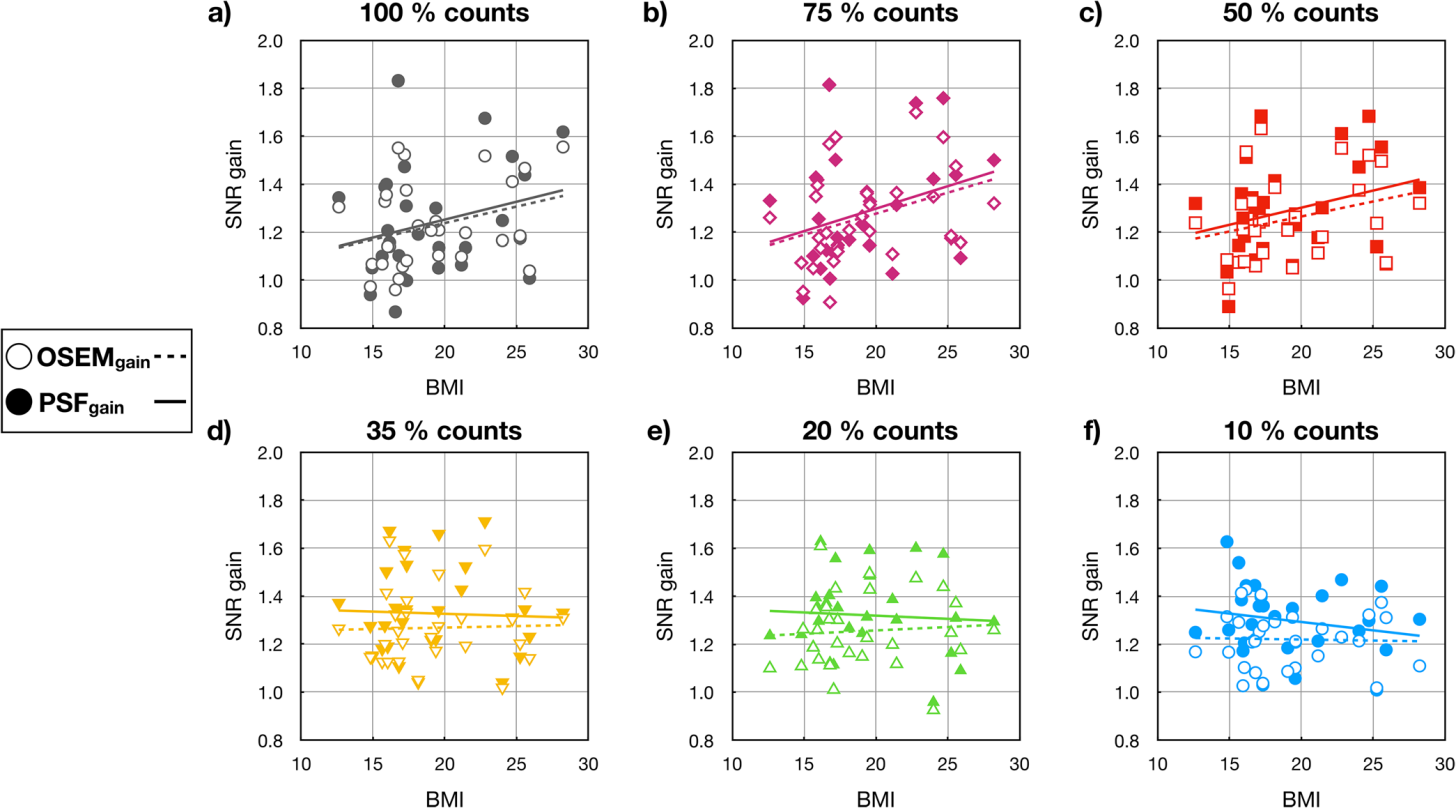
SNR gain, measured in the liver as a function of the BMI at all the count levels: 100, 75, 50, 35, 20 and 10% (**a**–**f**) of the original counts. Empty symbols correspond to OSEM SNR gain (OSEM only vs. TOF) and solid symbols to the PSF SNR gain (PSF vs. PSF+TOF). The gain in the SNR is higher when PSF is included in the reconstructions. The trend is shown as a linear regression with solid lines for PSF SNR gain reconstructions and dashed line for the OSEM SNR gain.

At 100% counts, the SNR gain changes, as a function of BMI, reached a maximum of 1.8 for the PSF reconstruction and 1.6 for OSEM only. At lower count levels, the trend was preserved, even when only 50% of the counts were used. Fig. 3 shows the SNR gain (liver) for OSEM only and PSF calculated for all the patients and plotted as a function of BMI for all count levels (10–100%). At count levels between 10 and 35%, the benefit of incorporating the TOF information was lost, independently of patient size.

The noise levels for the PSF+TOF reconstructions stayed below 20% when using only 35% of the counts (Suppl. Figure 4.).

**Fig. 4.**
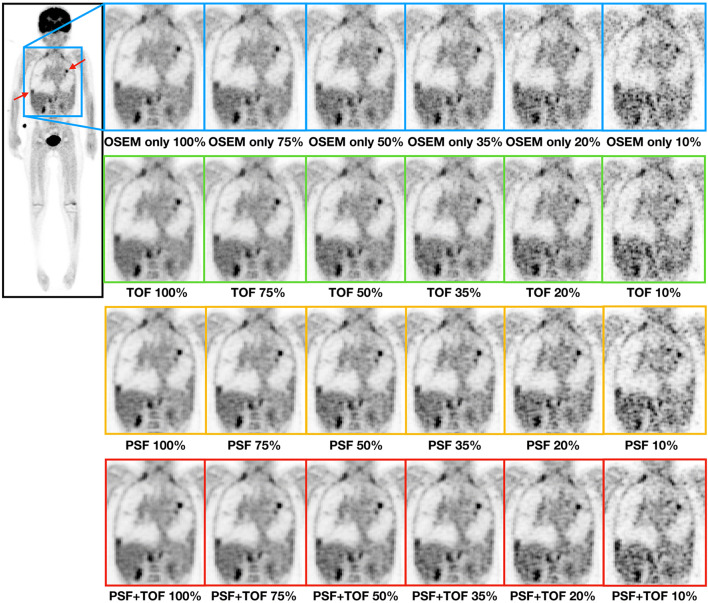
Comparison of the reconstructed images using OSEM only (blue), TOF (green), PSF (yellow) and PSF+TOF (red) at different count levels (100%, 75%, 50%, 35%, 20% and 10%). The images show a patient P21 (6-y/o, 21kg, BMI 17) with multiple lung tumours indicated with the arrows. No visual difference can be seen between the 100% and 75% images.

Fig. 4 shows sample images of a patient reconstructed with all 4 methods: OSEM only, TOF, PSF and PSF+TOF at all count levels. The visual differences across reconstruction methods at 100% of counts were minor; however, the images with added TOF expressed higher contrast and lower noise levels. When reducing the count levels with the OSEM only algorithm, the noise levels increased significantly, at 50% of the counts artefacts did appear in the liver and heart regions. These artefacts were not seen when the TOF information was included in the reconstructions. Nonetheless, low count images (10–35% counts) were generally not suitable for clinical analyses.

### Clinical Image Quality Assessment

**Fig. 5.**
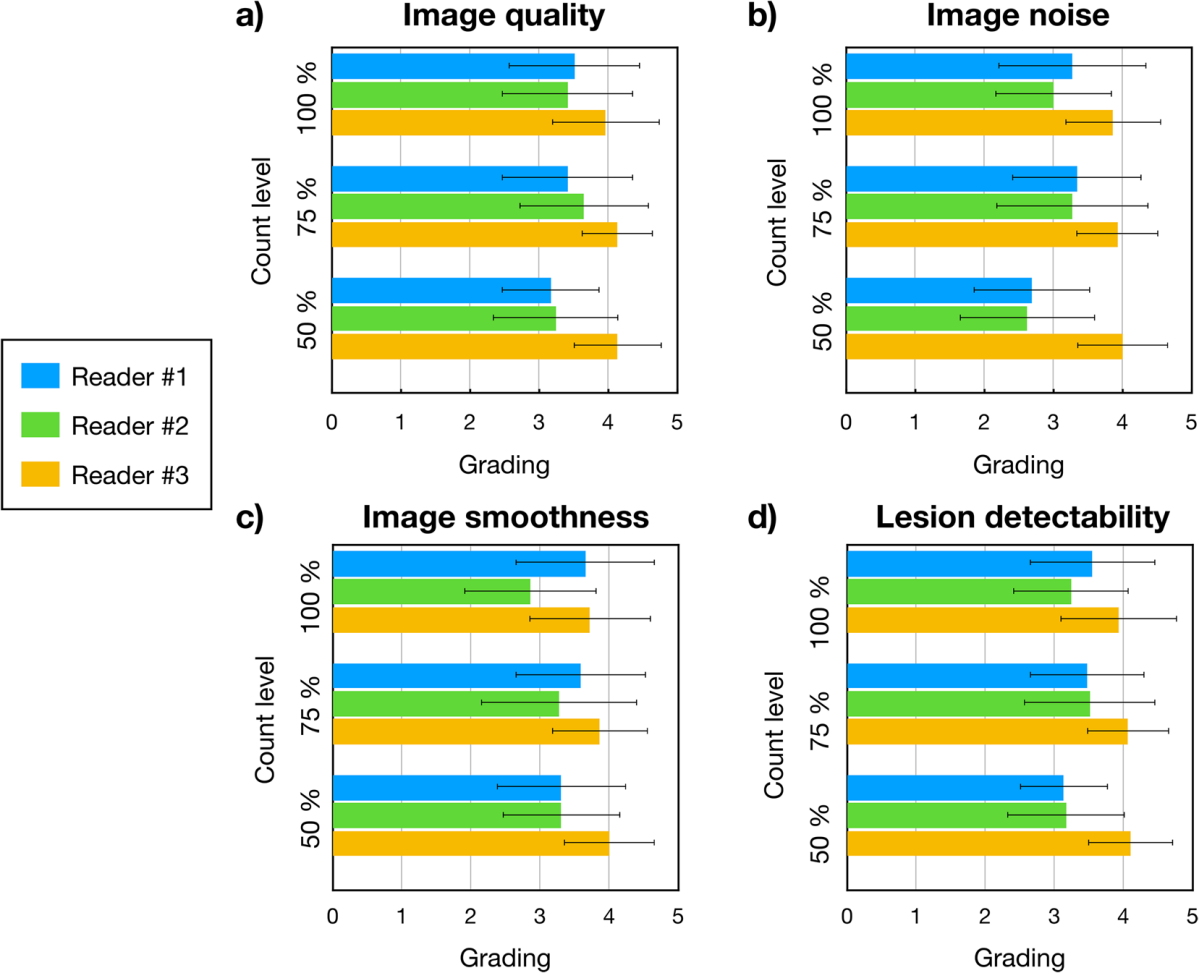
Bar plots of the mean values and standard deviations of the clinical readings for each reader at different count levels (50%, 75% and 100%) for the four quality questions.

Based on the initial visual and quantitative evaluation of the Nuclear medicine physicians, it was decided to employ the images reconstructed with PSF+TOF at 100, 75 and 50 of the original number of counts to assess the clinical image quality. Fig. 5 shows the mean and standard deviations values for the four quality responses across all patients. In general, all the readers were consistent with the level of grading. The third reader gave the highest gradings in all four categories. In terms of image quality, minor changes were seen when comparing the grading between 50–100% and 75–100%.

With the simulated reduced injected activities, the increased noise levels were observed by readers 1 and 2 for the images reconstructed using 50% of the counts compared with 100%, with average scores of 2.7 ± 0.8 and 2.6 ± 1.0 compared with 3.3 ± 1.1 and 3.0 ± 0.8, respectively (Fig. 5b). On the other hand, for lesion detectability, reader 1 gave an average score 3.6 ± 0.9 for the 100% count images and only a slightly lower score of an average of 3.1 ± 0.6 at 50% (Fig. 5d). In contrast, the second reader scored lesion detectability for both 100 and 50% count images almost similar at about 3.2 ± 0.8. The third reader gave the highest average score to the images reconstructed with 50% of total counts in all four categories. The mean scores for each question for all three readers including the coefficient of correlation and the corresponding *p* values are listed in Table 2. The test showed a medium positive correlation between the scores for low count images in comparison with the original data sets (50 vs 100% and 75 vs 100%) ranging from 0.53 to 0.75. In general, higher correlations were seen for the comparison between 75 and 100% images than between 50 and 100% images.

**Table 2:** Mean score and standard deviations for each of the 4 quality questions averaged across 3 readers and combined score for all questions, at different count levels. For each question, the mean scores for 50% and 100% and 75% and 100% were compared. The coefficient of correlation (r) and the corresponding p values reported (GP: 0.1234 (ns), 0.0332 (*), 0.0021 (**), 0.0002 (***), < 0.0001 (****))

	Mean (Standard deviation)			Correlation coefficient (r)		Significantly different?	
	50 %	75 %	100 %	50% vs 100%	75% vs 100%	50% vs 100%	75% vs 100%
Image quality	3.5 (0.6)	3.7 (0.7)	3.6 (0.7)	0.53	0.65	0.003**	0.0002***
Noise	3.1 (0.6)	3.5 (0.7)	3.4 (0.6)	0.58	0.58	0.001***	0.001***
Image smoothness	3.5 (0.6)	3.6 (0.7)	3.4 (0.6)	0.57	0.75	0.0012**	< 0.0001****
Lesion detectability	3.5 (0.5)	3.7 (0.6)	3.6 (0.6)	0.47	0.65	0.0097**	0.0001***
All	3.4 (0.9)	3.6 (0.9)	3.5 (0.9)	0.54	0.66	< 0.0001****	< 0.0001****

To further understand the significance of the changes between the count levels, the inter-reader agreement was analysed for the four categories (Table 3). The highest correlation was calculated for reader 1 and the lowest for reader 3. Only in the case of reader 1, for image quality and image noise was observed a higher positive correlation between 50 and 100% images than between 75 and 100%.

**Table 3:** Calculated coefficients of variation (r) and corresponding p values (GP: 0.1234 (ns), 0.0332 (*), 0.0021 (**), 0.0002 (***), < 0.0001 (****)) for each question for the individual clinical readers. Slight correlation was calculated for reader 1 and no correlation has been observed for reader 3

	Reader #1		Reader #2		Reader #3	
	50% vs 100%	75% vs 100%	50% vs 100%	75% vs 100%	50% vs 100%	75% vs 100%
Coefficient of correlation (r)						
Image quality	0.66	0.43	0.25	0.41	−0.06	0.37
Noise	0.57	0.33	0.26	0.50	−0.08	0.06
Image smoothness	0.31	0.33	0.36	0.40	0.00	−0.06
Lesion detectability	0.54	0.58	0.24	0.42	−0.05	0.22
*p* values						
Image quality	0.0001***	0.0201*	0.1861	0.0276*	0.7499	0.0494*
Noise	0.0012**	0.0797	0.1748	0.0057**	0.6848	0.7459
Image smoothness	0.1038	0.0790	0.0526	0.0311*	> 0.9999	0.7398
Lesion detectability	0.0025**	0.0009***	0.2039	0.0216*	0.7791	0.2423

### Injected Activity Levels

**Fig. 6.**
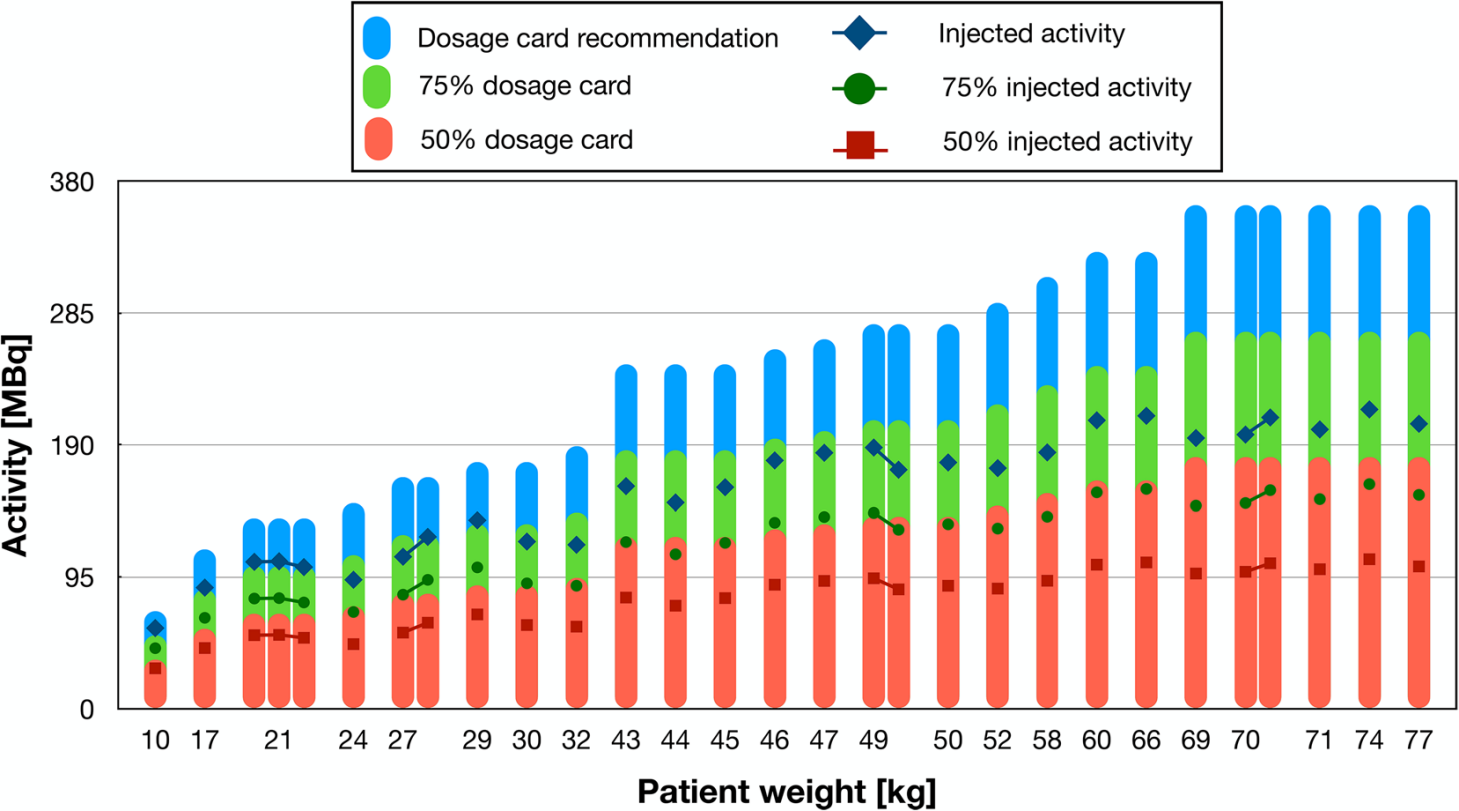
Comparison of the injected activity levels to the EANM recommendations for the analysed patients. The weight-based recommended injected activities for whole-body [18F]FDG imaging are shown as blue bars in the background. The injected activity of the paediatric patients is below 75% of the recommended activity for a [18F]FDG torso examination.

The injected and virtually reduced 18F[FDG] activity level for all patients was compared with the recommendation of the EANM paediatric dosage card (Fig. 6). Patients with a weight between 10 and 30 kg received an activity corresponding to ~ 75% of the recommendations. For larger patients, above 30 kg, the injected activity levels fall below 75% of the recommendations. When the collected counts were reduced to simulate 75% of the original injected activity (Fig. 6. green dots), this is corresponded to ~ 50% of the dose card recommendation.

## Discussion

We have assessed the effect of injected dose reduction when performing clinical 18F[FDG] whole-body imaging in paediatric oncology patients. We investigated the benefit of TOF at low count levels and evaluated reconstructed (PSF+TOF) PET images, both quantitatively and qualitatively. Based on the findings, the injected activity appears able to be reduced to 75% of the actual injected activity levels without significantly compromising image quality and lesion detectability.

Clinically viable iterative algorithms can lead to noisy images due to over iteration, as shown in previous studies [31]. The additional corrections added to the image reconstruction, such as resolution modelling (PSF) and TOF, lead to faster convergence of the reconstructions (Fig, 1). This behaviour is consistent with earlier findings [13, 26]. Ideally, the reconstruction parameters should be selected for every patient and clinical application; however, this is clinically not feasible. Therefore, reconstruction settings are usually optimized for a standard case. For example, reconstruction settings can be determined based on standard phantom acquisition (e.g., NEMA Image Quality phantom) [13]. In this study, a few patients were picked randomly from the cohort with different lesions and analysed to determine the optimal reconstruction parameters. This was done only for the original amount of count levels since the changes in the statistical distribution of the counts in the virtually reduced data sets have shown to have a negligible effect on the convergence of the PET reconstruction [32]. Following the initial analyses, in this study we used 5 iterations and 14 subsets for the non-TOF (OSEM only and PSF) and 3 iterations and 14 subsets when TOF information was included in the reconstructions (TOF and PSF+TOF).

The TOF-based SNR gain showed an expected trend with BMI for both OSEM only and PSF reconstruction; however, due to the induced image noise by the low counting statistics, the benefit on TOF was not seen at count levels below 50%. Increases in BMI had less of an influence on CNR gain, which can be associated with two reasons: first, the paediatric patients are significantly smaller compared with adults, with the highest BMI 28 kg/m^2^ from the analysed patient cohort, which corresponds to an average adult, and secondly, the influence of BMI on the SNR as well as CNR gain is more pronounced for small lesions in abdominal regions or head and neck and less noticeable in other examined areas. This behaviour is reasonable for the abdominal region as this region usually has the largest body extent and the TOF gain is dependent on the object diameter [13]. However, for the TOF gain in the head and neck region, no explanation could be found. Thus, more detailed studies have to be performed separating the different examination areas.

Three nuclear medicine physicians rated the images using a 5-point scale, answering four questions regarding the quality of the presented images. The Pearson’s correlation test showed a medium positive correlation across all the evaluated measures (0.58–0.75) between the 75 and 100% count images and a correlation between 50 and 100% of 0.47–0.58 (Table 2). On an individual basis, the highest correlation was seen for the readings by reader 1, whereas no correlations were seen for reader 3 (Table 3). Based on the calculated *p* values for the individual readings, the correlation was significant for 10 of the 24 score comparisons for the individual readers. Taking into account that originally injected activity levels correspond already to about ~ 75% of the recommendations by the EANM dosage card, our results indicate a possibility to reduce the injected activity by 50% of the EANM recommendations without significantly compromising image quality and lesion detectability. This reduction would translate into a 3 MBq/kg injection scheme following the Northern American definition for the injected activity compared to actual (5.2 - 3.7 MBq/kg).

The EANM paediatric dosage card was introduced in 2008 [6, 7]. In 2011, the baseline activity for the [18F]FDG PET torso examination was defined as 26 MBq following the critical suggestions of Holm et al. [5]. This minimum injected activity value was adopted also by the North American consensus recommendation in 2014 [8]. However, none of these studies took into consideration the specifications of the used PET system [8]. With further evaluations on the newly introduced PET systems, the reduction of the injected activity for 18F[FDG] whole-body paediatric PET/CT would appear feasible.

### Limitations of the Study

The main limitation of the study is the small number of paediatric patients with different indications, preventing a full evaluation of the tumours located in different regions. The reduction of the injected activity up to 75% of the administered dose was shown to be feasible on the basis of the presented patient cohort (Table 1); however, the results may be different when dealing with other patient groups with different disease status. Furthermore, new PET/CT systems have been recently introduced into the market equipped with silicon photomultiplier tubes and larger axial field-of-view, thus providing better TOF timing resolution and increased system sensitivity [9]. With these new technical advancements, the injected activities could be conceivably further reduced.

## Conclusion

We demonstrate that a significant reduction of administered activity by up to 75% is clinically feasible for 18F[FDG] whole-body paediatric PET/CT examinations without compromising lesion detectability for a state-of-the-art PET/CT systems with resolution recovery (PSF) and a 555ps TOF resolution.

## Supplementary Information

ESM 1 Combination of image reconstruction techniques and all simulated count levels for every analysed patient. Five new list-mode data sets were created with reduced amounts (10%, 20%, 35%, 50% and 75% from the original). The raw data was reconstructed with OSEM only and PSF image reconstructions and the reconstructions were repeated with added TOF information (TOF and PSF+TOF). For every reconstruction a 5mm FWHM Gaussian post-filter was added. All the count level and image reconstruction combinations were leading to 24 reconstructed data sets per patient. (JPG 975 kb)

ESM 2 General scheme of the applied virtual dose reduction method for random deletion of events in the original list-mode data. For every event is the LM data a random number is assigned (Selection of counts). Based on the selected value the event is deleted or kept in the newly generated LM data. The figure represents a virtual reduction of the count to 50%. (PNG 649 kb)

ESM 3 Schematic of the clinical evaluation: a) the reconstructed images were mixed and sorted into three reading days (Day 1, Day 2 and Day 3). Every day a patient was shown only once in a different order at a different count level (50%, 75% and 100% of the original counts); b) The readers had to answer four questions for every patient giving a grade on a 5-point scale; c) The 5-point scale where 5 is the best (optimal) and 1 is the worst grade (very poor/unacceptable). (PNG 564 kb)

ESM 4 Calculated noise levels (calculated in the liver) for all count levels (100%, 75%, 50%, 35%, 20% and 10%) and image reconstruction combinations (OSEM only, TOF, PSF, PSF+TOF). The lowest noise levels could be achieved with the PSF+TOF reconstructions and could be maintained below 20% when using only 35% of the counts as well. (JPG 1034 kb)
