# Supplementary figures and images for: Reducing Radiation Exposure to Paediatric Patients Undergoing [18F]FDG-PET/CT Imaging
Source: Mol Imaging Biol. 2021 Apr 12;23(5):775–86. doi: 10.1007/s11307-021-01601-4 (PMC8410733; doi:10.1007/s11307-021-01601-4)

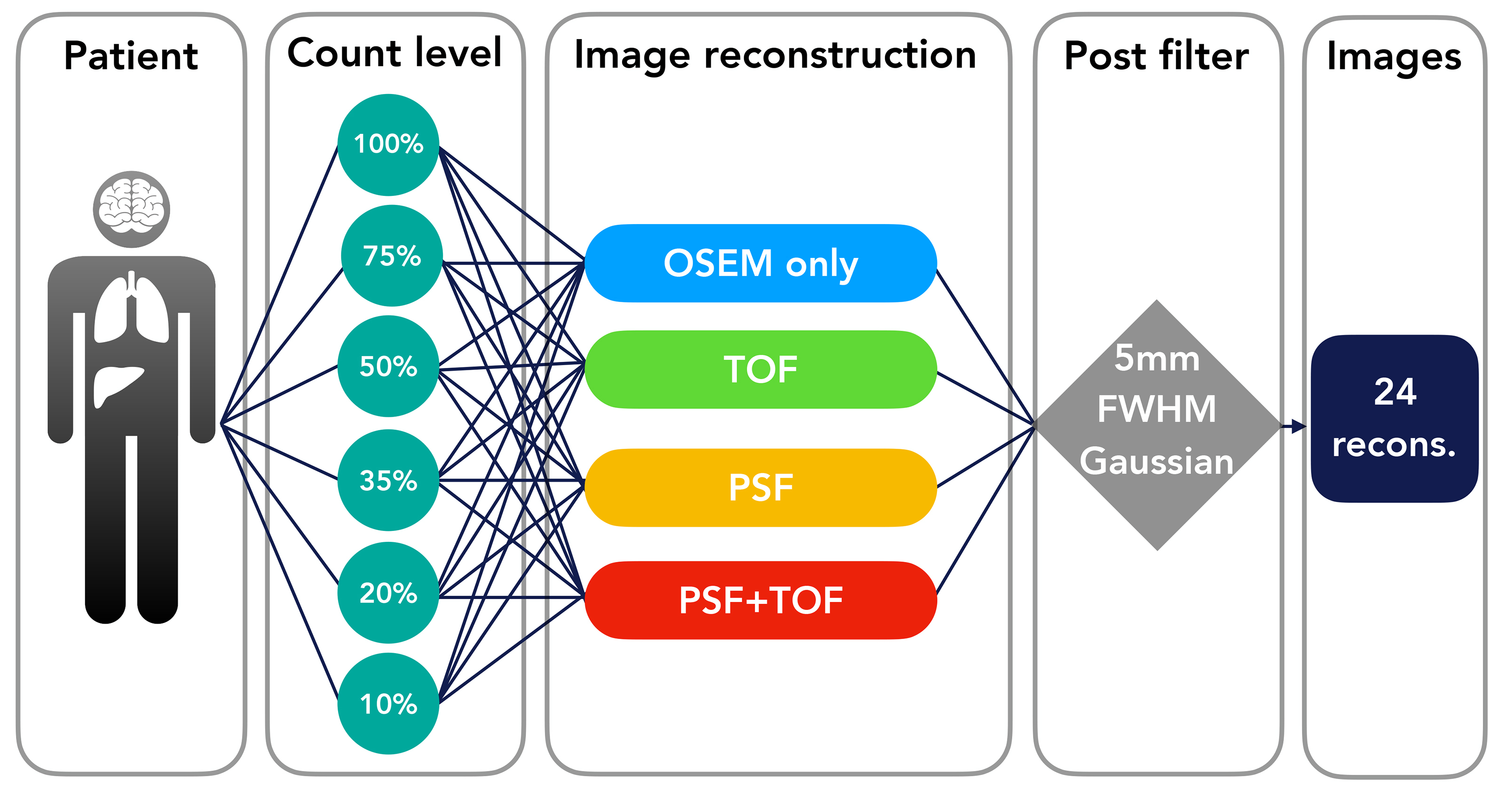

Supplement: Supplementary file 1 — Combination of image reconstruction techniques and all simulated count levels for every analysed patient. Five new list-mode data sets were created with reduced amounts (10%, 20%, 35%, 50% and 75% from the original). The raw data was reconstructed with OSEM only and PSF image reconstructions and the reconstructions were repeated with added TOF information (TOF and PSF+TOF). For every reconstruction a 5mm FWHM Gaussian post-filter was added. All the count level and image reconstruction combinations were leading to 24 reconstructed data sets per patient. (JPG 975 kb) [file 11307_2021_1601_MOESM1_ESM.jpg]

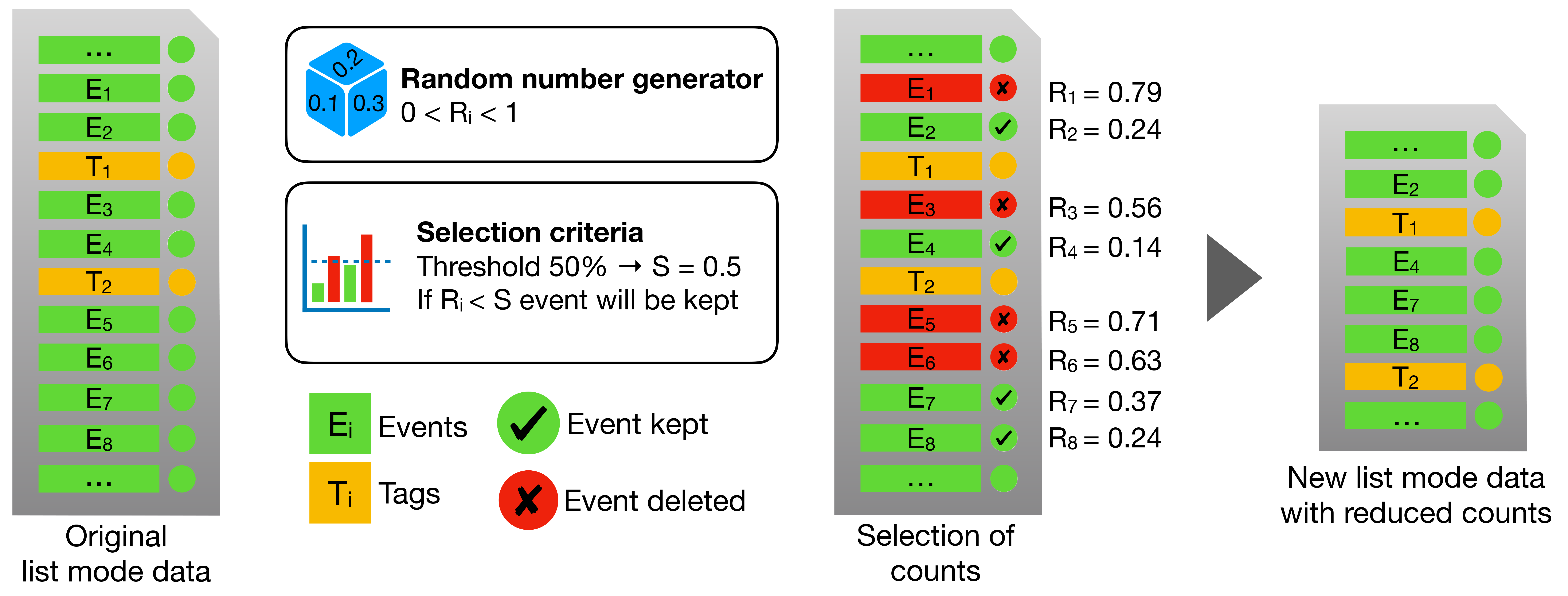

Supplement: Supplementary file 2 — General scheme of the applied virtual dose reduction method for random deletion of events in the original list-mode data. For every event is the LM data a random number is assigned (Selection of counts). Based on the selected value the event is deleted or kept in the newly generated LM data. The figure represents a virtual reduction of the count to 50%. (PNG 649 kb) [file 11307_2021_1601_MOESM2_ESM.png]

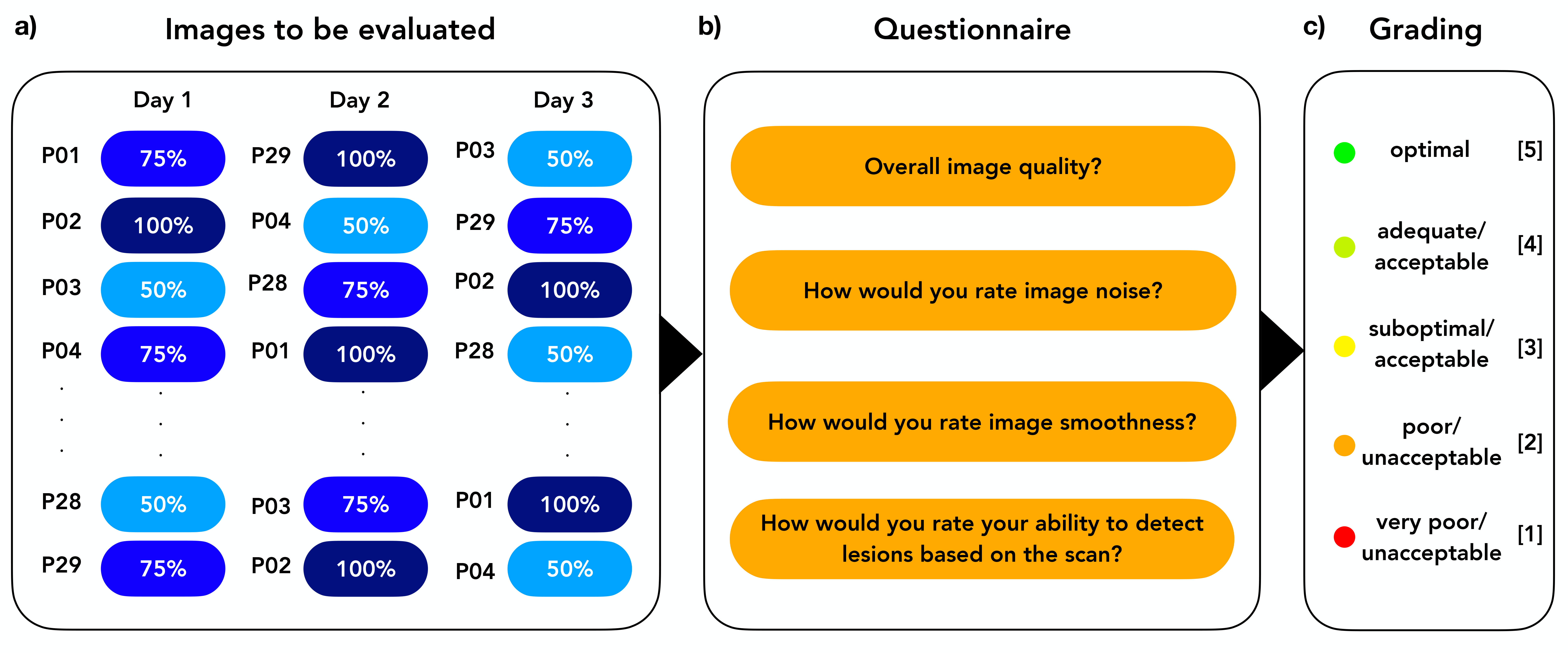

Supplement: Supplementary file 3 — Schematic of the clinical evaluation: a) the reconstructed images were mixed and sorted into three reading days (Day 1, Day 2 and Day 3). Every day a patient was shown only once in a different order at a different count level (50%, 75% and 100% of the original counts); b) The readers had to answer four questions for every patient giving a grade on a 5-point scale; c) The 5-point scale where 5 is the best (optimal) and 1 is the worst grade (very poor/unacceptable). (PNG 564 kb) [file 11307_2021_1601_MOESM3_ESM.png]

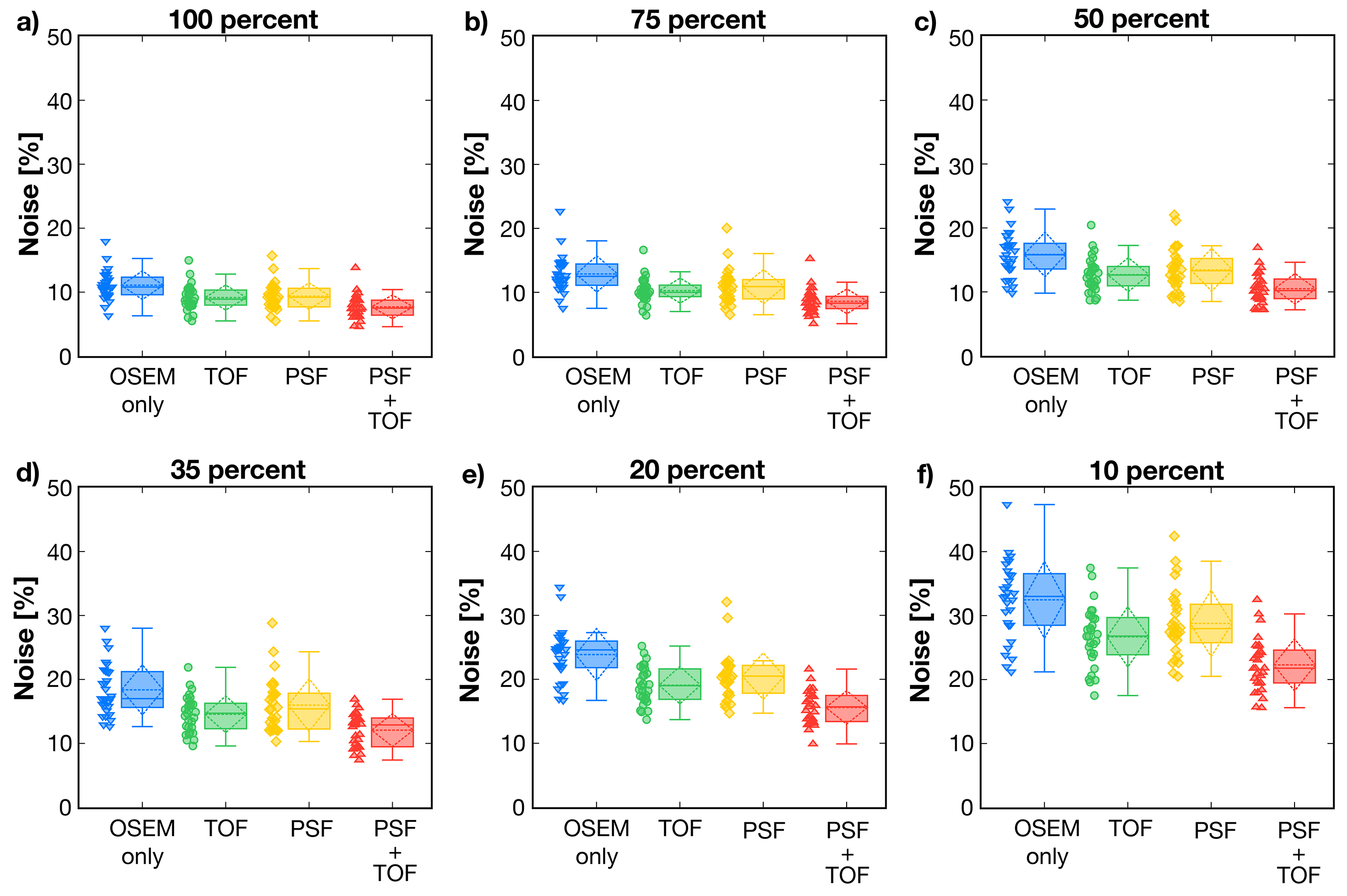

Supplement: Supplementary file 4 — Calculated noise levels (calculated in the liver) for all count levels (100%, 75%, 50%, 35%, 20% and 10%) and image reconstruction combinations (OSEM only, TOF, PSF, PSF+TOF). The lowest noise levels could be achieved with the PSF+TOF reconstructions and could be maintained below 20% when using only 35% of the counts as well. (JPG 1034 kb) [file 11307_2021_1601_MOESM4_ESM.jpg]
